# Plumbagin inhibits the proliferation and survival of esophageal cancer cells by blocking STAT3-PLK1-AKT signaling

**DOI:** 10.1038/s41419-017-0068-6

**Published:** 2018-01-16

**Authors:** Ying-Ya Cao, Jing Yu, Ting-Ting Liu, Kai-Xia Yang, Li-Yan Yang, Qun Chen, Feng Shi, Jia-Jie Hao, Yan Cai, Ming-Rong Wang, Wei-Hua Lu, Yu Zhang

**Affiliations:** 1 State Key Laboratory of Molecular Oncology, National Cancer Center/Cancer Hospital, Chinese Academy of Medical Sciences and Peking Union Medical College, 100021 Beijing, China; 2 Department of Intensive Care Medicine, Yijishan Hospital, Wannan Medical College, 241001 Wuhu, China

## Abstract

Esophageal squamous cell carcinoma (ESCC) is one of the deadliest cancers, and it requires novel treatment approaches and effective drugs. In the present study, we found that treatment with plumbagin, a natural compound, reduced proliferation and survival of the KYSE150 and KYSE450 ESCC cell lines in a dose-dependent manner in vitro. The drug also effectively inhibited the viability of primary ESCC cells from fresh biopsy specimens. Furthermore, plumbagin-induced mitotic arrest and massive apoptosis in ESCC cells. Notably, the drug significantly suppressed the colony formation capacity of ESCC cells in vitro and the growth of KYSE150 xenograft tumors in vivo. At the molecular level, we found that exposure to plumbagin decreased both polo-like kinase 1 (PLK1) and phosphorylated protein kinase B (p-AKT) expression in both ESCC cell lines. Enforced PLK1 expression in ESCC cells not only markedly rescued cells from plumbagin-induced apoptosis and proliferation inhibition but also restored the impaired AKT activity. Furthermore, signal transducer and activator of transcription 3 (STAT3), a transcription factor of PLK1, was also inactivated in plumbagin-treated ESCC cells; however, the overexpression of a constitutively activated STAT3 mutant, STAT3C, reinstated the plumbagin-elicited blockade of PLK1-AKT signaling in ESCC cells. Taken together, these findings indicate that plumbagin inhibits proliferation and potentiates apoptosis in human ESCC cells in vitro and in vivo. Plumbagin may exert these antitumor effects by abrogating STAT3-PLK1-AKT signaling, which suggests that plumbagin may be a novel, promising anticancer agent for the treatment of ESCC.

## Introduction

Esophageal squamous cell carcinoma (ESCC) is one of the most common malignancies worldwide and the fourth leading cause of cancer-related deaths in China^1,2^. ESCC is an aggressive malignant disease with a poor prognosis, and its treatment remains a significant challenge^3^. Few currently available drugs are effective in patients with ESCC; thus, potent anticancer agents are urgently needed.

Plumbagin (5-hydroxy-2-methyl-1,4-napthoquinone, PL), a natural quinoid constituent isolated from the roots of the medicinal plant *Plumbago zeylanica* L., exhibits striking anticancer activity in various human cancers, including lung, breast, ovarian, prostate, and colon cancer^4–11^. It has been shown that plumbagin can suppress malignant activity of cancer cells through multiple mechanisms, such as the inhibition of growth, invasion, and metastasis; induction of apoptosis; and anti-angiogenesis^4,5,7–10^. However, the antitumoral efficacy of plumbagin in ESCC remains to be determined.

In this work, we evaluated the potential antitumor effects of plumbagin against ESCC cells in vitro and in vivo. Furthermore, we explored the underlying mechanisms of these effects in ESCC cells.

## Results

### Plumbagin inhibits the proliferation of ESCC cells

**Fig. 1:**
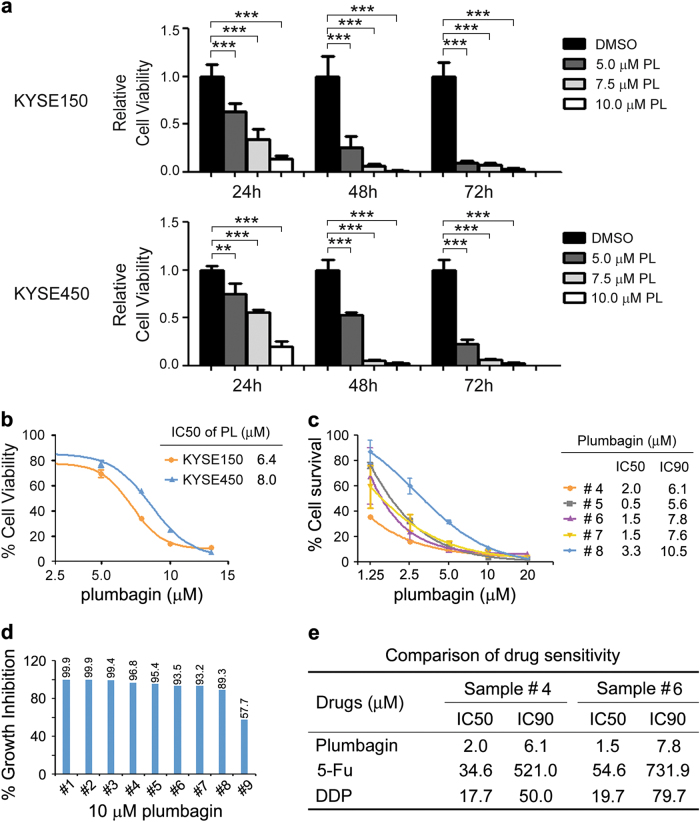
Plumbagin inhibits cell proliferation in ESCC cells **a**,**b** ESCC cell line KYSE150 and KYSE450 were treated with increasing concentrations of plumbagin for 24–72 h. Cell viability was determined with a CCK-8 assay. **a** The effects of plumbagin on cell viability of ESCC cells. Data are presented as mean ± SEM (*n* = 3). **P < 0.01, ***P < 0.001. **b** ESCC cells were treated with plumbagin for 24 h, and the IC_50_ was calculated. **c**,**d** Primary ESCC cells cultured in vitro were treated with plumbagin for 72 h, and cell viability were assessed using ATP-TCA. **c** Primary ESCC cells from five patients were treated with increasing doses of plumbagin. **d** Primary ESCC cells from nine patients were treated with 10 μM plumbagin. **e** Comparison of drug sensitivity in primary ESCC cells from two patients

To explore the potential cytotoxicity of plumbagin to ESCC cells, we first assessed the effect of plumbagin on cell proliferation. The treatment of two independent ESCC cell lines, KYSE150 and KYSE450, with various concentrations of plumbagin significantly inhibited cell proliferation in a concentration-dependent and time-dependent manner (Fig. 1a). The IC_50_ values of plumbagin in KYSE150 and KYSE450 cells were 6.4 ± 0.2 and 8.0 ± 0.3 μM, respectively (Fig. 1b).

To further examine the antitumor efficacy of plumbagin in clinical specimens, we performed a chemosensitivity assay using primary ESCC cells isolated from fresh biopsy tissues. Exposure to plumbagin for 72 h inhibited the proliferation of ESCC cells in a concentration-dependent manner, and the average IC_50_ and IC_90_ values of plumbagin in tested samples were 1.8 ± 0.4 and 7.5 ± 0.9 μM (*n* = 5), respectively (Fig. 1c). In addition, 10 μM of plumbagin reduced proliferation by ∼90% in 89% (8/9) of biopsy specimens (Fig. 1d), indicating that most of the ESCC cells responded well to the drug. Moreover, we compared the efficacy of plumbagin with two drugs currently used in ESCC chemotherapy, 5-Fu and Cisplatin (DDP). Notably, in the samples from two ESCC patients, the IC_50_ and IC_90_ of plumbagin were markedly lower than those of 5-Fu and DDP (Fig. 1e), indicating that plumbagin is more effective compared to either 5-Fu or DDP in these cases.

### Plumbagin induces cell cycle arrest and apoptosis in ESCC cells

**Fig. 2:**
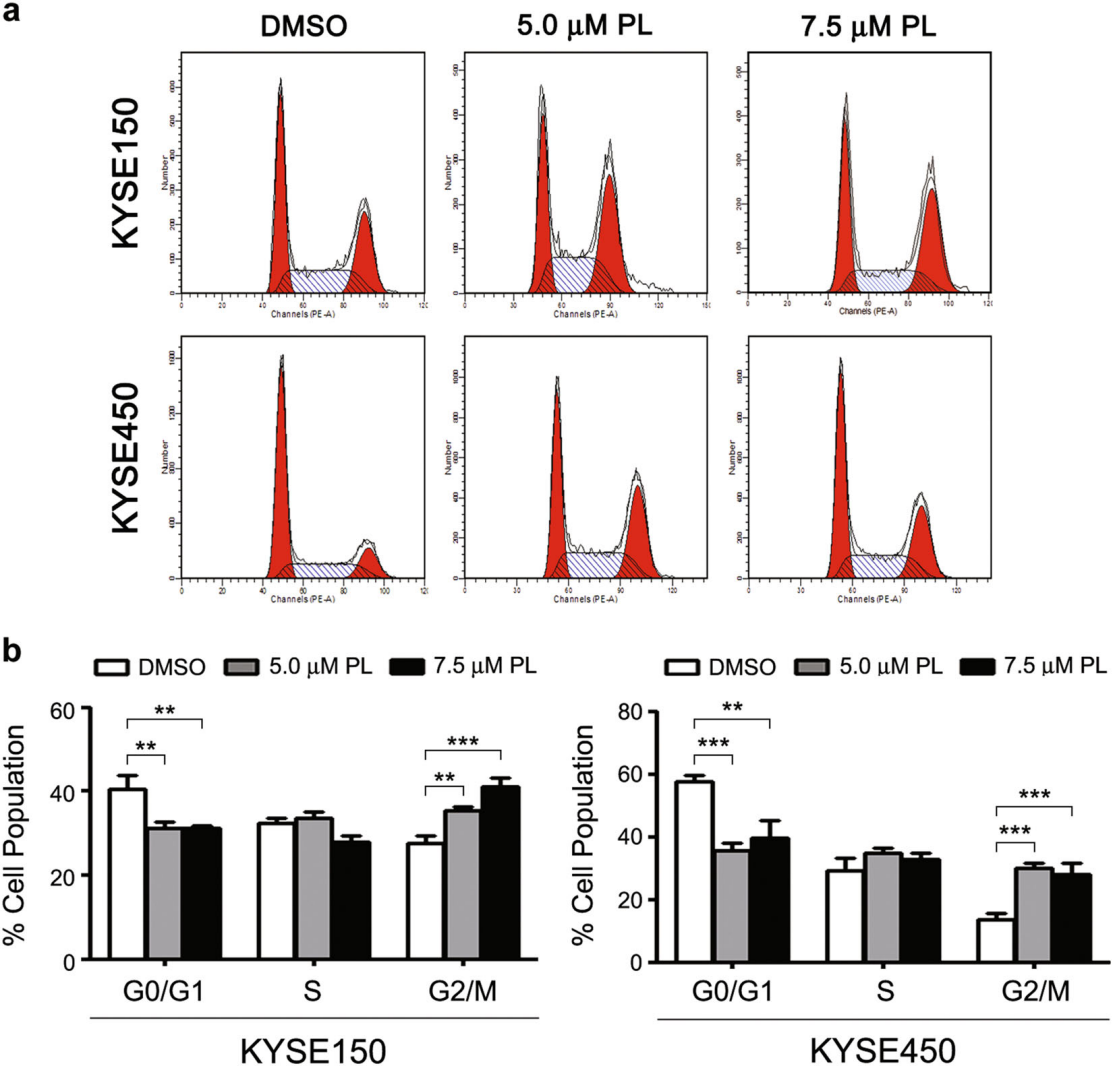
Plumbagin arrests the progression of the cell cycle in ESCC cells KYSE150 and KYSE450 cells were treated with 5 or 7.5 μM of plumbagin for 12 h, and the cell cycle distribution was then assessed using flow cytometry. **a** Representative results of the cell cycle analysis. **b** Histogram of cell cycle distribution. Data are represented as mean ± SEM (*n* = 3). **P < 0.01, ***P < 0.001

**Fig. 3:**
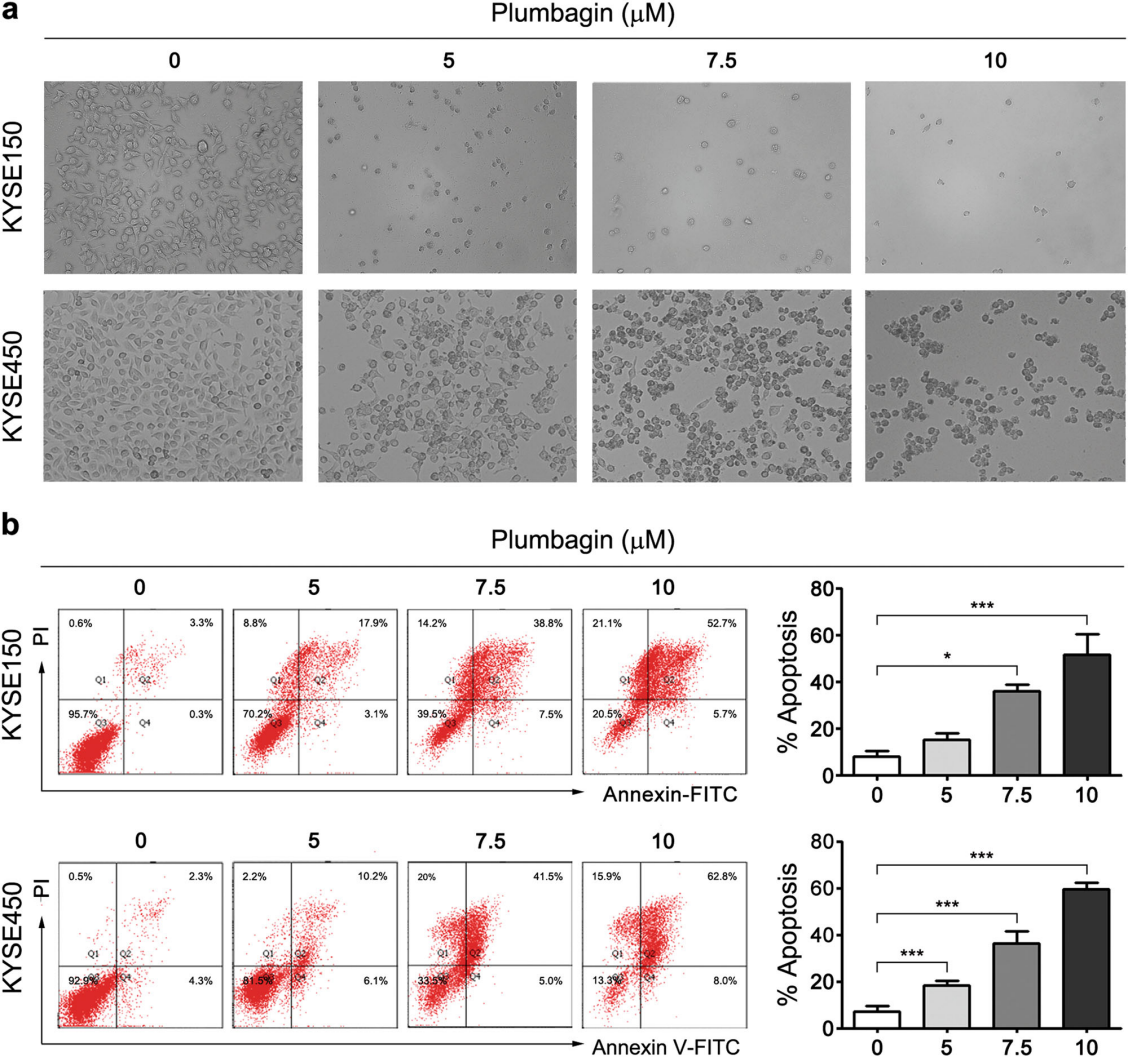
Plumbagin promotes apoptosis in ESCC cells KYSE150 and KYSE450 cells were incubated with 0, 5, 7.5, or 10 μM plumbagin for 24 h. **a** Representative images of cells treated with plumbagin obtained with a phase contrast microscope. Scale bar = 100 μm. **b** Cell apoptosis was assessed based on Annexin V-FITC/PI double staining and flow cytometry. Representative results are shown and percentage of apoptotic cells was plotted. Data are represented as mean ± SEM (*n* = 3). *P<0.05, ***P < 0.001

To decipher the mechanisms underlying the plumbagin-mediated inhibition of proliferation, we analyzed the cell cycle distribution using flow cytometry. Following 12 h of plumbagin treatment, the number of KYSE150 and KYSE450 cells in the G2/M phase significantly increased, which was accompanied by a marked decrease of cells in the G0/G1 phase (Fig. 2a and b, Supplementary Table 1). In addition, obvious changes in apoptotic morphology were observed in KYSE150 and KYSE450 cells 24 h after exposure to plumbagin (Fig. 3a), and this plumbagin-induced apoptosis was dose-dependent in both cell lines (Fig. 3b).

### Plumbagin suppresses the tumorigenicity of ESCC cells

**Fig. 4:**
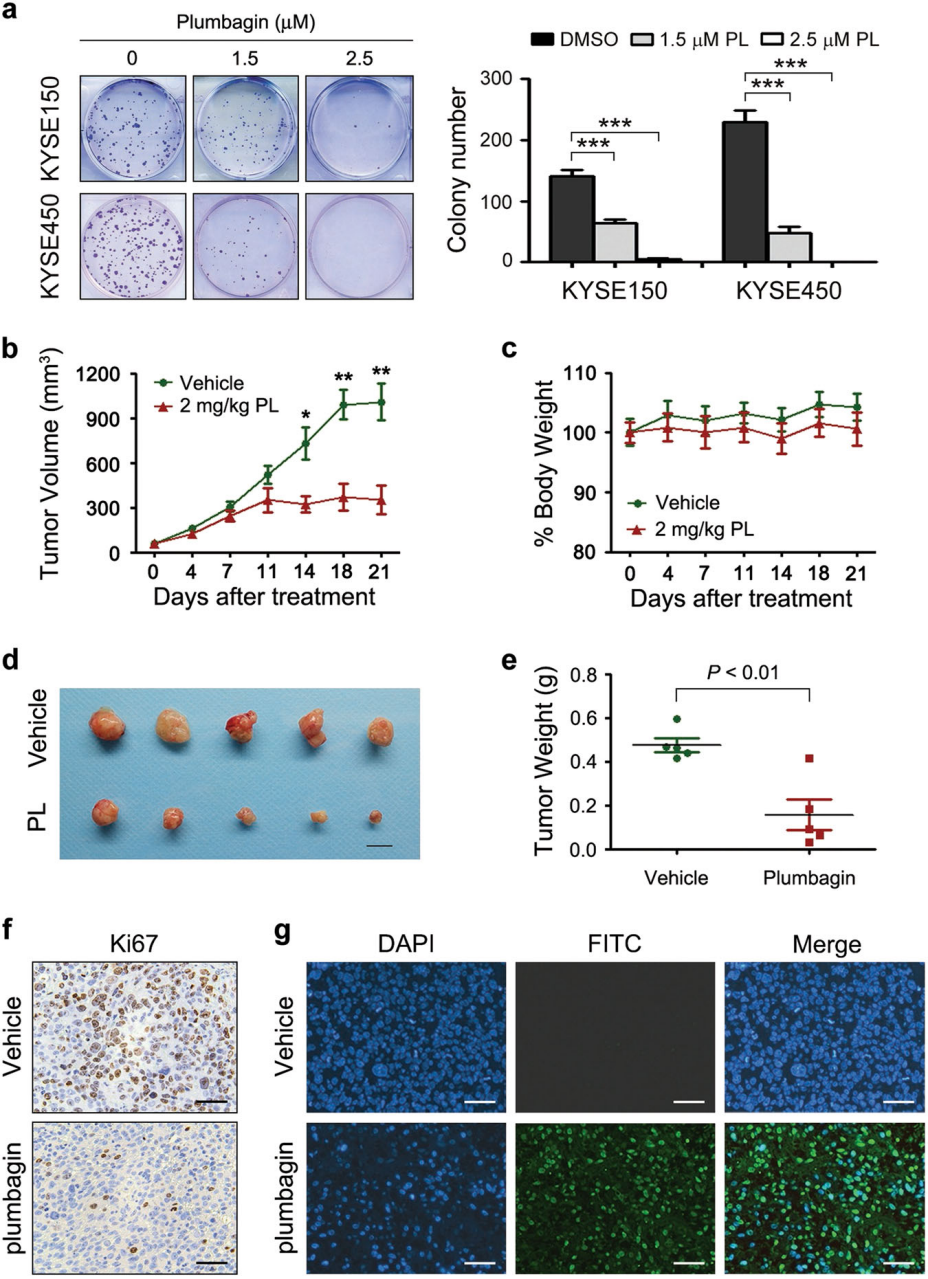
Plumbagin suppresses colony formation and xenograft tumor growth **a** Colony formation assays with ESCC cells exposed to the indicated concentrations of plumbagin (PL) for 6 h. Representative results are shown, and the number of colonies is plotted. Data are presented as mean ± SEM (*n* = 3). ***P < 0.001. **b**–**g** Nude mice bearing established KYSE150 xenograft tumors (~50 mm^3^) were i.p. injected with 2 mg/kg plumbagin or vehicle control 5 days per week for 3 weeks. The tumor volumes **b** and body weight of mice **c** were measured at the indicated time after plumbagin administration. Data are presented as mean ± SEM (*n* = 5). Body weight is expressed as a percentage relative the initial weight at the beginning of treatment. *P < 0.05, **P < 0.01. **d** Xenograft tumors. Scale bar = 1 cm. **e** Comparison of tumor weights. **f** Intratumoral proliferation was assessed based on Ki67 immunostaining. Bar = 50 μm. **g** Apoptosis was measured with a TUNEL assay. Bar = 50 μm

Subsequently, we investigated the effects of plumbagin on the tumorigenicity of ESCC cells. First, we observed that plumbagin markedly and dose-dependently suppressed the colony formation capacity of KYSE150 and KYSE450 cells (Fig. 4a). Then, xenograft tumors derived from KYSE150 cells were utilized to evaluate the antitumor efficacy of plumbagin in vivo, since the xenograft tumors derived from KYSE450 cells were mainly comprised of necrotic tissues. The intraperitoneal (i.p.) administration of plumbagin (2 mg/kg/day, 5 days per week for 3 weeks) drastically attenuated the growth of subcutaneous (s.c.) KYSE150 xenograft tumors in nude mice, as determined by marked decreases in tumor volume compared with vehicle-treated controls (Fig. 4b). However, plumbagin treatment did not result in observable weight loss during the experimental period (Fig. 4c), indicating that the drug lacks apparent systemic toxicity in vivo. The weights of plumbagin-treated xenograft tumors were also significantly lower than those of control tumors (Fig. 4d, e). In accordance with the strong antitumor effect of plumbagin observed in vivo, treatment with this drug also evidently inhibited proliferation and enhanced apoptosis in xenograft tumors, as evidenced by Ki67 immunostaining and TUNEL assays performed on s.c. tumor sections (Fig. 4f, g).

### PLK1 is a pivotal cellular target of plumbagin in ESCC cells

**Fig. 5:**
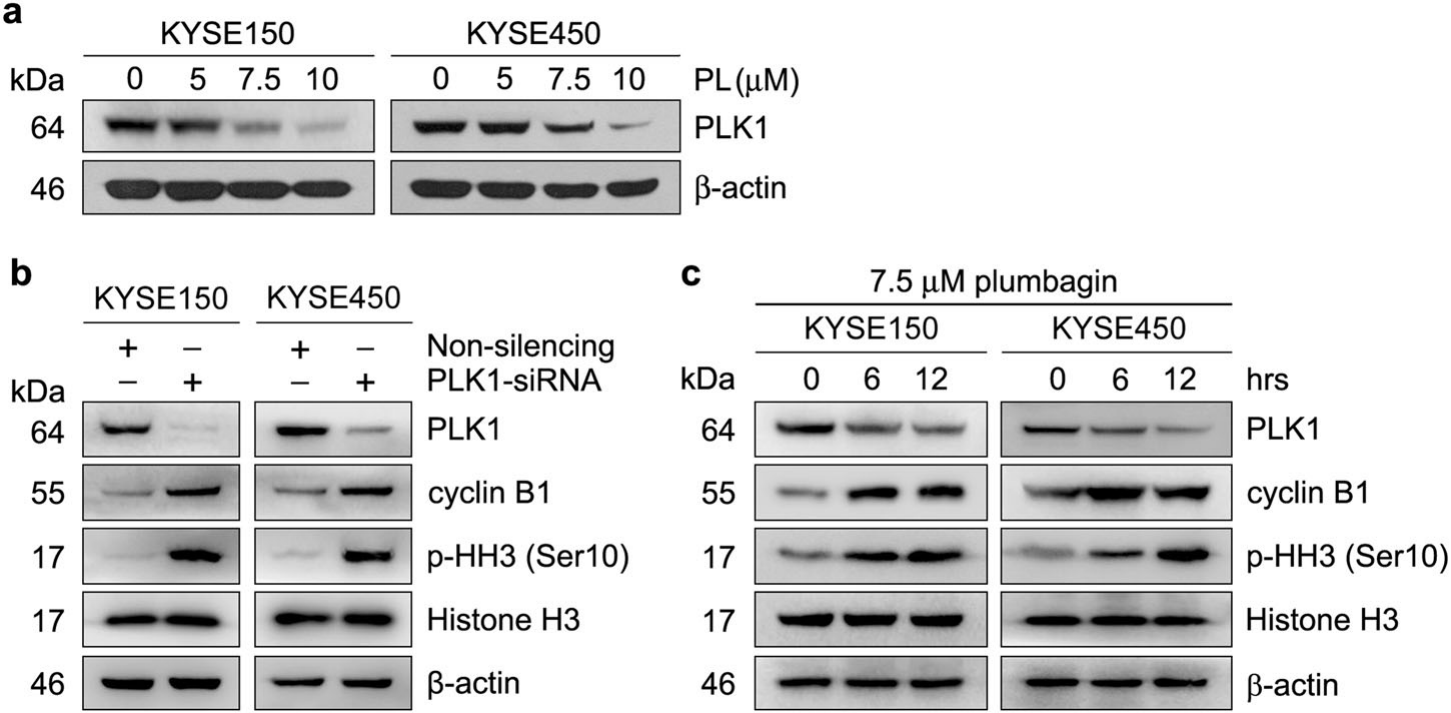
PLK1 downregulation contributes to plumbagin-mediated mitotic arrest **a** KYSE150 and KYSE450 cells were treated with increasing concentrations of plumbagin (PL) for 24 h. **b** KYSE150 and KYSE450 cells were transiently transfected with PLK1 siRNA or control non-silencing siRNA for 48 h. **c** KYSE150 and KYSE450 cells were treated with 7.5 μM plumbagin for the indicated times. Cell lysates were immunoblotted for the indicated proteins. β-actin was used as a loading control

Our previous studies have demonstrated that the overexpression of PLK1 plays an essential role in the proliferation and apoptosis resistance of ESCC cells^12,13^. Specifically, depletion of PLK1 with a specific siRNA resulted in marked G2/M arrest and drastic apoptosis in KYSE150 and KYSE450 cells (Supplementary Fig. 1); plumbagin induced similar effects. Hence, we speculated that plumbagin may suppress cell proliferation and survival by suppressing PLK1 expression in ESCC cells. To test this hypothesis, we examined changes in PLK1 expression in plumbagin-treated ESCC cells. Indeed, exposure to plumbagin for 24 h reduced the PLK1 protein levels in KYSE150 and KYSE450 cells in a dose-dependent manner, indicating that plumbagin is a potent inhibitor of PLK1 (Fig. 5a).

PLK1 is a key regulator of cell division^14^. It has been reported that PLK1 inhibition efficiently arrested cells in mitosis by preventing cyclin B1 degradation at anaphase^15^. The present study demonstrated that PLK1 depletion resulted in a mitotic block with a concomitant increase in the levels of cyclin B1 and phospho-Histone H3 (Ser10), a mitosis marker, in both ESCC cell lines (Supplementary Fig. 1a and b, Fig. 5b). In parallel, the levels of cyclin B1 and p-Histone H3 (Ser10) were also elevated in plumbagin-treated ESCC cells (Fig. 5c), implying that plumbagin-mediated PLK1 downregulation prevents cyclin B1 degradation, which contributes to drug-induced mitotic arrest.

**Fig. 6:**
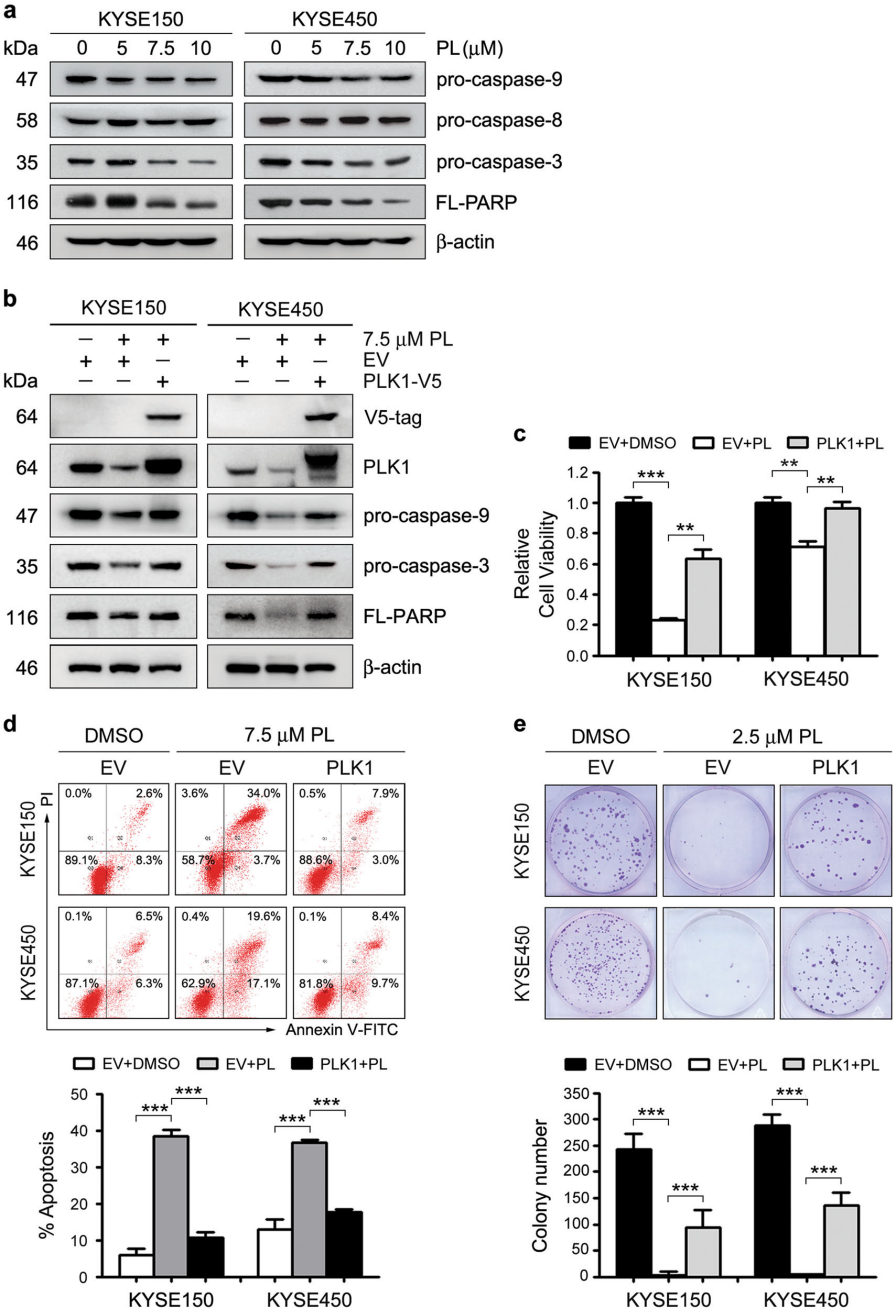
PLK1 downregulation is required for plumbagin-induced proliferation inhibition and apoptosis **a** KYSE150 and KYSE450 cells were treated with plumbagin for 24 h, and the levels of indicated proteins were assessed by Western blotting. β-actin was used as a loading control. **b-e** The inducible stable clone strains KYSE150-TO-PLK1-V5 and KYSE450-TO-PLK1-V5 cells were pre-treated with 1 μg/ml doxycyclin for 24 h to induce exogenous PLK1 expression, and then treated with the indicated dose of plumbagin (PL) for 24 h. Cells stably transfected with Empty vector (EV) were used as controls. **b** Cell lysates were immunoblotted for the indicated proteins. **c** Cell viability was assessed using a CCK-8 assay. **d** Apoptosis were analyzed with Annexin V-FITC double-staining and flow cytometry. The representative results are shown and percentages of apoptotic cells were plotted. **e** Colony formation assays of cells exposed to the indicated concentrations of plumbagin. Representative results are shown and colony number was quantified. **c-e**. Data are represented as mean ± SEM (*n* = 3). **P < 0.01; *** *P* < 0.001

In most cancer cells, mitotic arrest induced by PLK1 inhibition triggers apoptosis^14,16–18^. Our earlier work showed that PLK1 depletion lead to apoptosis by activating the mitochondrial apoptotic pathway in ESCC cells^13^. As with PLK1 knockdown, treatment of ESCC cells with plumbagin induced the cleavage of pro-caspase-9, but not pro-caspase-8 in KYSE150 and KYSE450 cells. Meanwhile, the levels of pro-caspase-3 and full-length poly (ADP-ribose) polymerase (PARP), a cleavage target of caspase-3, were reduced upon drug treatment (Fig. 6a). Moreover, enforced PLK1 expression partially restored the plumbagin-induced cleavage of pro-caspase-9, pro-caspase-3, and full-length PARP in both cell lines (Fig. 6b). Collectively, the data indicated that plumbagin-elicited PLK1 downregulation potentiates apoptosis by activating the intrinsic mitochondrial apoptotic pathway in ESCC cells.

Remarkably, ectopically expressed PLK1 attenuated the plumbagin-mediated inhibition of proliferation, survival, and colony formation in both ESCC cell lines compared to control cells. This further demonstrated that plumbagin-induced PLK1 downregulation largely imparts antitumor activity in ESCC cells (Fig. 6c–e).

### Plumbagin inactivates AKT by downregulating PLK1

**Fig. 7:**
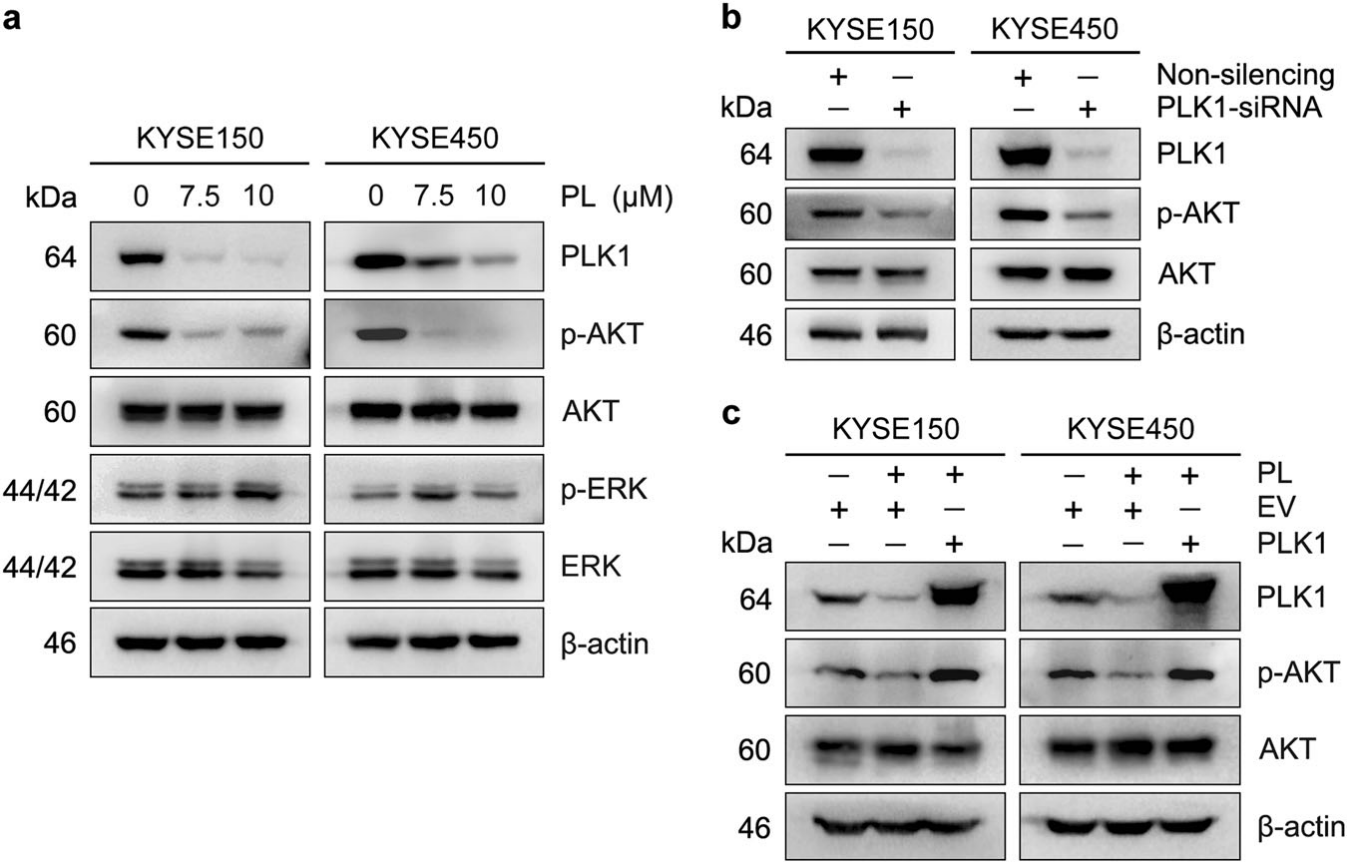
Plumbagin suppresses AKT activity through downregulating PLK1 **a** ESCC cells were treated with 0, 7.5 or 10 μM plumbagin (PL) for 24 h. **b** ESCC cells were transiently transfected with PLK1 siRNA or non-silencing control siRNA for 48 h. **c** PLK1 overexpressed stable clone cells or empty vector (EV) control cells described in Fig. 6 were treated with 7.5 μM plumbagin (PL) for 24 h. Cell lysates were immunoblotted for the indicated proteins. β-actin was used as a loading control.

Next, we identified critical downstream signaling pathways modulated by plumbagin in ESCC cells. Among the pathways that are closely associated with cell proliferation and survival, plumbagin treatment markedly suppressed PI3K-AKT signaling in KYSE150 and KYSE450 cells, as demonstrated by a reduction in the p-AKT levels (Fig. 7a). Likewise, PLK1 knockdown abrogated the activation of AKT (Fig. 7b). Moreover, exogenous PLK1 overexpression effectively reversed this reduction in the AKT levels (Fig. 7c).

### STAT3 is involved in the plumbagin-mediated blockade of PLK1-AKT cascade

**Fig. 8:**
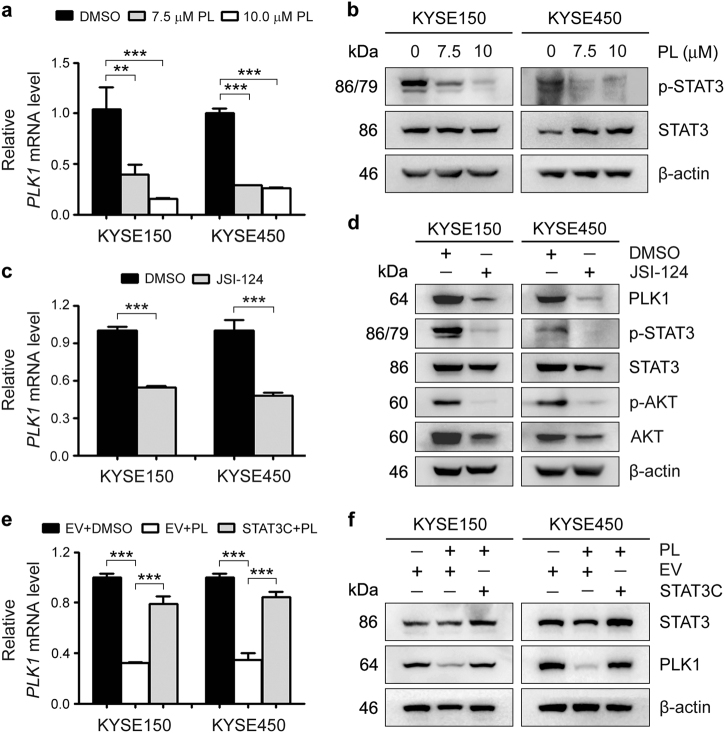
Plumbagin blocks PLK1-AKT signaling by inactivating STAT3 **a**,**b** KYSE150 and KYSE450 cells were treated with plumbagin (PL) for 24 h. **a** The PLK1 mRNA levels were examined by qRT-PCR. GAPDH was used as an internal reference. **b** The activity of STAT3 were assessed by Western blotting. β-actin was used as a loading control. **c**,**d** Cells were treated with 10 μM JSI-124 for 24 h, and the PLK1 mRNA and indicated protein levels were then measured with qRT-PCR **c** and Western blotting **d**. **e**,**f** ESCC cells stably transfected with pBabe-Stat3C or empty vector were treated with 7.5 μM plumbagin (PL) for 24 h, and the mRNA or protein level of PLK1 was measured with qRT-PCR **e** and Western blotting **d**. **a**,**c** and **e** Data are represented as mean ± SEM (*n* = 3). **P < 0.01; ***P < 0.001

Finally, we explored the molecular mechanism underlying the plumbagin-induced downregulation of PLK1. Exposure to plumbagin drastically decreased the PLK1 mRNA levels, implying that plumbagin influences PLK1 transcription (Fig. 8a). Accordingly, earlier studies have shown that plumbagin can inhibit STAT3 activity^4,19–21^. In addition, we previously demonstrated that STAT3 can transcriptionally activate PLK1 expression in ESCC cells. Therefore, we herein examined changes in STAT3 activity in plumbagin-treated ESCC cells. As expected, plumbagin treatment decreased the p-STAT3 levels (Fig. 8b). In addition, blocking JAK2/STAT3 signaling with a highly selective inhibitor JSI-124 (cucurbitacin I) distinctly downregulated PLK1 at both the mRNA and protein levels, and these changes were accompanied by diminished AKT activity (Fig. 8c, d). Moreover, the overexpression of a constitutively activated STAT3 mutant, STAT3C, markedly restored the reduction of PLK1 expression mediated by plumbagin (Fig. 8e, f). These data suggest that plumbagin may inhibit ESCC tumor growth by abrogating the STAT3-PLK1-AKT cascade.

## Discussion

ESCC is a common cancer with poor prognosis, and is unresponsive to most conventional chemotherapeutic agents. We show here that plumbagin effectively suppresses the proliferation and survival of ESCC cells in vitro and in vivo. Furthermore, our data indicate that the plumbagin-mediated tumor-inhibitory effects are closely associated with the inhibition of STAT3-PLK1-AKT pathway in ESCC cells.

Although plumbagin has exhibited potent antitumor activity in several cancer types^10^, its impact on ESCC cells remains unclear. Our present work revealed that plumbagin has strong antitumor efficacy against ESCC cells in culture and in mice, which is consistent with its effects on some other cancer types^4,7,22–24^. In addition, our xenograft assay showed that the effective dose of plumbagin (2 mg/kg, i.p.) had no apparent systemic toxicity in vivo, reminiscent of an earlier finding that plumbagin did not markedly influence the viability of normal cells, i.e., peripheral blood mononuclear cells, even at 100 µM^6^. Particularly, we confirmed the growth-inhibitory effect of plumbagin in primary ESCC cells from biopsy tissues. Cisplatin (DDP) and 5-Fu are currently used in patients with unresectable locally advanced or metastatic ESCC; however, their antitumor efficacy and response rate are not satisfactory^3^. Notably, ∼90% of ESCC biopsy specimens were sensitive to plumbagin, even in DDP and 5-Fu insensitive cases. Altogether, our data suggest that plumbagin is a novel promising agent for ESCC therapy.

To date, the mechanisms underlying the anticancer action of plumbagin are not fully understood^10^, and the cellular targets of plumbagin in ESCC cells remain unidentified. In the present study, we found that plumbagin treatment resulted in pronounced G2/M arrest and apoptosis in ESCC cells, which contributes to its antitumor effects. PLK1 is a member of the highly conserved serine/threonine protein kinase family, and it is a key regulator of cell division^25–27^. We previously reported that PLK1 was overexpressed in ∼70% of ESCC tissues. Furthermore, suppression of PLK1 expression or activity significantly inhibited the proliferation and survival of ESCC cells in culture and in mice^12,13,28^. PLK1 depletion and plumbagin treatment resulted in identical phenotypes (i.e. G2/M arrest and apoptosis), which raises the possibility that plumbagin suppresses cell proliferation and survival by regulating PLK1 expression in ESCC cells. As expected, plumbagin treatment decreased PLK1 expression. Moreover, overexpression of exogenous PLK1 prominently decreased the inhibitory potency of plumbagin. Collectively, these data reveal that PLK1 is an essential target of plumbagin, and the cytotoxicity of plumbagin to ESCC cells is at least partially due to downregulation of PLK1.

PLK1 is a cell cycle-related kinase required for proper M-phase progression. PLK1 inhibition leads to pronounced mitotic arrest followed by apoptosis^14,16–18^. Plumbagin treatment also arrested ESCC cells in the mitosis phase, as indicated by the elevated cyclin B1 and p-Histone H3 (Ser10) levels. Cyclin B1 is an important regulator of mitosis. At the end of mitosis (anaphase), cyclin B1 is degraded by the anaphase-promoting complex (APC), thereby permitting cells to exit mitosis. PLK1 has been shown to activate the APC by phosphorylating several of its components. The activated APC catalyzes the polyubiquitination of cyclin B1 and causes its degradation^15^. Altogether, our results imply that plumbagin-mediated PLK1 downregulation lead to aberrant cyclin B1 accumulation at anaphase, thereby arresting the cells in mitosis. In contrast, plumbagin-induced earlier G2/M arrest in some cancer types, which was associated with decreased amounts of cyclin B1^29–31^. These different findings imply that the effects of the drug may be cell type-specific or cell context-specific, which may be largely dependent on its cellular targets and associated signaling pathway. Furthermore, our work demonstrated that plumbagin activated the intrinsic mitochondrial apoptotic pathway by impairing PLK1 expression in ESCC cells.

Previous reports have shown that plumbagin can inhibit cell proliferation and survival by modulating multiple signaling pathways^4,9,10,21,32^. However, the impact of plumbagin on the PLK1-related network is not clear. PLK1 is known to be an upstream kinase of the AKT^33,34^, and we found that both plumbagin treatment and PLK1 depletion inactivated AKT in ESCC cells. More importantly, enforced PLK1 expression markedly restored AKT activity in plumbagin-treated ESCC cells, suggesting that PLK1 is a critical mediator of plumbagin-induced AKT inactivation. Moreover, our data also suggest that plumbagin directly downregulates PLK1 mRNA expression by abolishing the activity of STAT3, a transcriptional factor of PLK1. Intriguingly, a bioinformatics analysis predicted that plumbagin can inactivate STAT3 by directly binding to the DNA-binding domain of STAT3 and packing against its residues, such as Gly-421, Gly-419, Cys-418, Arg-417, Glu-415, and Arg-382^35^, implying that STAT3 is a direct cellular target of plumbagin. Taken together, this work provides evidence that plumbagin can exert its antitumor effects by abrogating STAT3-PLK1-AKT signaling, thus revealing a novel anticancer mechanism of the drug.

## Conclusions

Here, we report that plumbagin elicits potent antitumor activity against ESCC by reducing cell proliferation and survival in vitro and in vivo. We further identify the STAT3-PLK1-AKT signaling pathway as a pivotal molecular target of plumbagin in ESCC cells. Overall, our results suggest that plumbagin is a promising anticancer drug for the treatment of ESCC, which build a foundation for the clinical trials of this novel therapeutic agent.

## Materials and methods

### Tumor tissues

ESCC biopsy tissues form nine patients were collected at Chinese Academy of Medical Sciences and Peking Union Medical College (CAMS & PUMC), Beijing, China. The clinicopathological features of biopsy specimens is described in Supplementary Table 1. None of the patients had received chemotherapy of radiotherapy. All patients signed separate informed consent forms for sampling and research. This study was approved by the Ethics Committee/Institutional Review Board of National Cancer Center/Cancer Hospital, CAMS and PUMC (No. NCC2013RE-025).

### Cell culture and treatments

The human ESCC cell lines KYSE 150 and KYSE 450 were gifts from Dr. Y. Shimada (Kyoto University, Kyoto, Japan). The background information of both cell lines is provided in Supplementary Table 2. The cell lines were cultured in RPMI 1640 medium supplemented with 10% fetal bovine serum (Invitrogen, San Diego, CA), penicillin (100 U/ml), and streptomycin (100 mg/ml) at 37 °C under 5% CO_2_ in a humidified incubator. ESCC cells were incubated with plumbagin (Sigma, St. Louis, MO) at the indicated concentrations and for the indicated times. Vehicle (DMSO)-treated cells were used as controls.

### Cell viability assay

Cells were seeded in triplicate in 96-well plates at a density of 8000 cells per well. After an overnight incubation, the cells were exposed to different concentrations of plumbagin for the indicated times. Cell viability was assessed with a Cell Counting kit-8 (CCK-8, Dojindo, Japan) according to the manufacturer’s protocol. The absorbance at 450 nm was measured using an ELX808 microplate spectrophotometer (BioTek Instruments, Winooski, VT, USA).

### Colony formation assay

To assess the clonogenic capacity of cells in vitro, 500 cells were plated in six-well culture plates in triplicated. Following a 24 h incubation, the cells were treated with plumbagin for 6 h. After being washed with PBS, the cells were allowed to grow for 10–14 days to form colonies, and then fixed with methanol and stained with crystal violet. The number of colonies was counted.

### Cell cycle analysis

Cells were seeded at a density of 5 × 10^5^ per well in six-well plates and incubated at 37 °C overnight. The cells were treated with plumbagin (0, 5 and 7.5 μM) for 12 h and then collected by trypsinization and fixed in ice-cold 70% ethanol overnight at 4 °C. The cell cycle distribution was analyzed using a Cell cycle detection kit (Nanjing KeyGen Biotech, Nanjing, China) and BD^TM^ LSRΙΙ flow cytometer (BD Biosciences, San Jose, CA, USA). The resultant cell cycle profiles were analyzed using the ModFit software (Verity Software House, Topshem, ME, USA).

### Apoptosis detection

The morphology of ESCC cells treated with plumbagin was observed under a phase-contrast microscope (Leica DMI4000B, Leica Microsystems Wetzlar GmbH, Wetzlar, Germany). Apoptotic cells were double-labeled with AnnexinV-FITC and PI using the Annexin V/FITC kit (Neo Bioscience, Beijing, China) and analyzed with a BD^TM^ LSRΙΙ flow cytometer (BD Biosciences). Annexin V-positive cells were calculated and defined as apoptotic cells. A TUNEL assay using the Dead-End Fluorometric TUNEL System (Promega, Madison, WI) was performed to detect intratumoral apoptosis as described previously[12].

### Chemosensitivity assay

Fresh ESCC tissue specimens were digested and separated to generate single-cell suspensions, which were seeded in 96-well plates at a density of 2–4 × 10^4^ cells per well. The primary cultured ESCC cells were then treated with plumbagin, 5-fluorouracil (5-Fu, Tianjin Kingyork Group Co., Ltd., Tianjin, China), or Cisplatin (DDP, Qilu Pharmaceutical Co., Ltd., Jinan, China) for 72 h. The viability of cells was assessed using an ATP bioluminescence tumor chemosensitivity assay (ATP-TCA). All essential materials and reagents, including the tissue dissociation enzyme, red blood cell lysis buffer, culture medium, ATP standard, ATP maximal inhibitor, luciferase–luciferin mix, and various buffers, were provided by the tumor chemosensitivity assay kit (Beijing Gold Amethyst Pharm & Biotech Co. Ltd., Beijing, China). Light units were measured using a microplate luminometer (BHP9504, Hamamatsu Photo Medical Techniques Inc., Beijing, China). The growth inhibition rate was calculated using the following formula: (1-absorption of experimental group/absorption of vehicle control) ×100%.

### Small interfering RNA and plasmid constructs

The following siRNA target sequence was used to silence human PLK1: 5′-AGATCACCCTCCTTAAATATT-3′^13^. A scrambled siRNA sequence, 5′-TTCTCCGAACGTGTCACGT-3′, was used as a negative control. The oligonucleotides were chemically synthesized by GenePharma (Shanghai, China). All plasmid constructs generation are described in the Supplementary materials.

### Transfection and lentiviral transduction

Cells were transfected with siRNA or plasmid vectors using Lipofectamine 2000 (Invitrogen) according to the manufacturer’s instructions. To generate the lentivirus, 293FT cells (Invitrogen) were cotransfected with psPAX2, pMD2.G, and either pLenti6.3-TO-PLK1-V5 or pLenti6.3-TO-EV. Forty-eight hours after transfection, the lentiviral supernatants were harvested and passed through a 0.45 μM filter. The lentiviruses were added to media supplemented with 8 µg/ml polybrene (Sigma) to transduce ESCC cells following the manufacturer’s instructions.

### Stable clone selection and induction of *PLK1* expression

To establish inducible PLK1-overexpressing ESCC cell strains, KYSE150 or KYSE450 cells were infected with pLenti6.3-TO-PLK1-V5 lentivirus and further selected for 10 days with 1.5 and 0.7 μg/ml blasticidin (Invitrogen), respectively, to generate stable pools named KYSE150-TO-PLK1 or KYSE450-TO-PLK1. Concurrently, KYSE150 or KYSE450 stable clones infected with pLenti6.3-TO-V5-EV, named KYSE150-TO-EV and KYSE450-TO-EV, were used as controls. To induce exogenous PLK1 expression in KYSE150-TO-PLK1 and KYSE450-TO-PLK1 cells in vitro, 1 μg/ml doxcycline (Dox, Selleckchem, Houston, TX, USA) was added to the culture medium. The expression of PLK1-V5 in stable clone cells was verified by Western blotting using V5 antibody.

### Western blot analysis

SDS-PAGE and Western blotting were performed according to standard protocols. Briefly, the protein lysates were separated on SDS-PAGE gels and then transferred onto PVDF membranes (Millipore, Bedford, MA, USA). The blots were blocked and probed with primary antibodies against PLK1 (Abcam, Cambridge, UK), V5 (Invitrogen), pro-caspase-3, full-length PARP (FL-PARP) (Proteintech, Wuhan, China), p-Histone-H3(p-HH3)(Ser10)(Immunoway Biotechnology, TX, USA), pro-caspase-8 (Atlas Antibody, Stockholm, Sweden), cyclin B1, pro-caspase-9, Histon-H3, p-ERK (Thr202/Tyr204), ERK1/2, p-AKT (Ser473), AKT, Stat3, and p-Stat3 (Tyr705) (Cell Signaling Technology, Danvers, MA, USA). β-actin (Amart, Shanghai, China) was used as a loading control. The signals were visualized with a super-enhanced chemiluminescence detection reagent (Applygen).

### Xenograft model antitumor assay

All animal procedures were approved by the Institutional Animal Care and Use Committee at the National Cancer Center/Cancer Hospital, CAMS & PUMC (No. NCC2013A014), and followed the National Institutes of Health guide for the care and use of Laboratory animals. KYSE150 cells were subcutaneously inoculated into the flanks of 4-week-old female nude mice (BALB/c-nu, HFK Bioscience, Beijing, China). A total of 1×10^6^ cells were injected per animal. When the tumor volume reached ∼50 mm^3^, the mice were randomly divided into two groups (*n* = 8) and treated daily for 5 days per week with plumbagin (2 mg/kg body weight, i.p.) prepared in 25% PEG or vehicle control. The mice were weighed and tumor size was measured twice weekly. The tumor volume (*V*) was calculated with the following formula: *V* = 0.524×*L*×*W*^2^, where *L* is the length and *W* is the width of the xenograft tumor. The mice were euthanized, and the average weight of tumor tissues was obtained after 3 weeks of treatment.

### Immunohistochemistry

Xenograft tumors from vehicle-treated and plumbagin-treated mice were fixed in 10% neutral buffered formalin, embedded in paraffin, and used to prepare 4 μM-thick sections. These slides were immunohistochemically stained with anti-Ki67 antibody (Cell Signaling Technology) as described previously^12^.

### Real-time quantitative RT-PCR

Total RNA isolation, cDNA synthesis and real-time quantitative RT-PCR (qRT-PCR) was performed as previously described [12]. GAPDH was used as an internal reference.

### Statistical analysis

Statistical analysis was performed using the SPSS 19.0 program (SPSS Inc., Chicago, IL). Experimental results were statistically evaluated using the Student’s *t*-test, a one-way ANOVA, or the nonparametric Kruskal–Wallis test. A *P*-value < 0.05 was considered significant.

## Electronic supplementary material

Supplementary table 1

Supplementary table 2

Supplementary Table 3

Supplementary materials

Supplementary Figure 1
