# Supplementary material for: Plumbagin inhibits the proliferation and survival of esophageal cancer cells by blocking STAT3-PLK1-AKT signaling
Source: Cell Death Dis. 2018 Jan 16;9(2):17. doi: 10.1038/s41419-017-0068-6 (PMC5833725; doi:10.1038/s41419-017-0068-6)
Supplement: Supplementary file 1 — Supplementary table 1 [file 41419_2017_68_MOESM1_ESM.docx]

**Supplementary table 1.** **Clinicopathological features of ESCC biopsy specimens from 9 patients**

| **Case No.** | **Age** | **Gender** | **TNM**  **Classification** | **Grade** | **Immunology** |
| --- | --- | --- | --- | --- | --- |
| 1 | 86 | male | - | - | - |
| 2* | 61 | male | pT3N1M0 | G2 | CyclinD1(1+), EGFR(2+), HER2(-), VEGF(1+), c-MET(1+) |
| 3* | 63 | male | pT3N2M0 | G2 | CyclinD1(3+), EGFR(3+), HER2(-), VEGF(-), c-MET(1+) |
| 4 | 58 | female | - | - | - |
| 5 | 61 | male | - | - | - |
| 6* | 64 | male | pT3N0M0 | G2/G3 | CyclinD1(1+), EGFR(3+), HER2(-), VEGF(-), c-MET(1+) P40(3+), P63(3+), CK18(2+), |
| 7 | 58 | male | - | - | - |
| 8 | 54 | male | - | - | - |
| 9 | 66 | male | - | - | - |

Note: -, no information was obtained.

*, Three patients received surgery and histopathologic examinations. Other specimens were diagnosed as ESCC by biopsy pathology.
