# Supplementary material for: Plumbagin inhibits the proliferation and survival of esophageal cancer cells by blocking STAT3-PLK1-AKT signaling
Source: Cell Death Dis. 2018 Jan 16;9(2):17. doi: 10.1038/s41419-017-0068-6 (PMC5833725; doi:10.1038/s41419-017-0068-6)
Supplement: Supplementary file 2 — Supplementary table 2 [file 41419_2017_68_MOESM2_ESM.docx]

**Supplementary table 2. The information of ESCC cell lines used in this study [1-4]**

|  | **KYSE150** | **KYSE450** |
| --- | --- | --- |
| DSMZ No. | ACC 375 | ACC 387 |
| Patient′s information | | |
| age | 49 | 59 |
| gender | female | Male |
| Histology Grade | G3 | G1 |
| Immunology | cytokeratin +, cytokeratin-7 -, cytokeratin-8 +, cytokeratin-17 +, cytokeratin-18 +, cytokeratin-19 -, desmin -, endothel -, EpCAM +, GFAP -, neurofilament -, vimentin - | cytokeratin +, cytokeratin-7 -, cytokeratin-8 +, cytokeratin-17 +, cytokeratin-18 +, cytokeratin-19 +, desmin -, endothel -, EpCAM +, GFAP -, neurofilament -, vimentin - |
| Cytogenetics | human hypertriploid karyotype with 12% polyploidy - 70(68-75)<3n>XderX, -X, +1, -3, -4, -8, -9, +11, -14, -15, -16, -18, -21, +12mar (4xbisat), add(X)(q27), inv(1)(p34q32), del(1)(p32), del(1)(q11), del(7)(q32), del(8)(p21), i(9q), add(9)(q34), add(11)(p11), der(11)add(11)(p1?)add(11)(q24), del(1)(p11), del(12)(q21), del(15)(q23q25), add(19)(q13) | human hyperdiploid karyotype with 6.7% polyploidy - 57(49-57)<2n>, -Y, +1, +3, +3, +7, +12, +17, -18, +7mar, der(1)t(1;6)(p22;q11), add(1)(p32/34), del(2)(p23), del(3)(p22), del(4)(q21), add(5)(q35), del(6)(q25), add(7)(p22), add(8)(p23), add(9)(q34), del(11)(q14q21), add(12)(q14), del(12)(p11p12), der(14)t(8;14)(q21;p11), del(17)(p11), der(19)t(1;19)(q11;q12) - sideline with add(11)(q?23) - extensive subclonal rearrangements of ch 7 |
| Major molecular aberrations | | |
| *TP53* | Mutation | Mutation |
| *EGFR* | WT | Mutation |
| *ALK* | WT | Mutation |
| *SMAD4* | WT | Mutation |
| *NOTCH1* | WT | Mutation |
| *NOTCH2* | WT | Mutation |
| *PTEN* | WT | WT |
| *ERBB3* | Mutation | WT |
| *PDGFRA* | Mutation | WT |
| *Cyclin D1* | Amplification (4×) | Single copy |
| *EGFR* | Amplification (8×) | Single copy |
| *MYC* | Single copy | Amplification (6×) |

Note：-, no information was obtained.

1. DSMZ Cell Culture Data (<https://www.dsmz.de/home.html>)

2. Kanda, Y., Nishiyama, Y., Shimada, Y., Imamura, M., Nomura, H., Hiai, H., and Fukumoto, M. (1994). Analysis of gene amplification and overexpression in human esophageal-carcinoma cell lines. Int J Cancer 58, 291-297.

3. Elkabets, M., Pazarentzos, E., Juric, D., Sheng, Q., Pelossof, R.A., Brook, S., Benzaken, A.O., Rodon, J., Morse, N., Yan, J.J., et al. (2015). AXL mediates resistance to PI3Kalpha inhibition by activating the EGFR/PKC/mTOR axis in head and neck and esophageal squamous cell carcinomas. Cancer Cell 27, 533-546.

4. Lin, D.C., Hao, J.J., Nagata, Y., Xu, L., Shang, L., Meng, X., Sato, Y., Okuno, Y., Varela, A.M., Ding, L.W., et al. (2014). Genomic and molecular characterization of esophageal squamous cell carcinoma. Nat Genet 46, 467-473.
