# Supplementary material for: Plumbagin inhibits the proliferation and survival of esophageal cancer cells by blocking STAT3-PLK1-AKT signaling
Source: Cell Death Dis. 2018 Jan 16;9(2):17. doi: 10.1038/s41419-017-0068-6 (PMC5833725; doi:10.1038/s41419-017-0068-6)
Supplement: Supplementary file 3 — Supplementary Table 3 [file 41419_2017_68_MOESM3_ESM.docx]

**Supplementary Table 3. Cell cycle distr****ibution in plumbagin-treated ESCC cells**

| Cell lines | treatment | G0/G1 (%) | S (%) | G2/M (%) |
| --- | --- | --- | --- | --- |
| KYSE150 | DMSO | 40.4 ± 1.822 | 32.2 ± 0.815 | 27.4 ± 1.139 |
|  | 5.0 μM PL | 31.2 ± 0.762 | 33.4 ± 0.921 | 35.5 ± 0.546 |
|  | 7.5 μM PL | 31.1 ± 0.406 | 27.9 ± 0.769 | 41.0 ± 1.139 |
| KYSE450 | DMSO | 57.6 ± 1.114 | 29.0 ± 2.236 | 13.4 ± 1.132 |
|  | 5.0 μM PL | 35.6 ± 1.225 | 34.7 ± 0.835 | 29.7 ± 0.929 |
|  | 7.5 μM PL | 39.5 ± 3.194 | 32.6 ± 1.241 | 27.9 ± 2.098 |

Note: PL, plumbagin.
