# Supplementary material for: Plumbagin inhibits the proliferation and survival of esophageal cancer cells by blocking STAT3-PLK1-AKT signaling
Source: Cell Death Dis. 2018 Jan 16;9(2):17. doi: 10.1038/s41419-017-0068-6 (PMC5833725; doi:10.1038/s41419-017-0068-6)
Supplement: Supplementary file 4 — Supplementary materials [file 41419_2017_68_MOESM4_ESM.docx]

**Supplementary materials**

**Plasmid Constructs**

Plasmid vectors of pBabe-Stat3C (constitutively activated Stat3 mutant) and pBabe were provided by Dr J. Bromberg as generous gifts (Memorial Sloan-Kettering Cancer Center, New York, NY).

The PLK1 coding sequence was amplified from pEGFP-PLK1(ref. 13 ) by PCR using the forward primer: 5′-ACTAGTCTGCAGGGAGCATGAGTGCTGCAGTGACTGCAGGGA-3′, and the reverse primer: 5′-CTCGAGGGAGGCCTTGAGACGGTTGCT-3′. PCR was performed using the Platinum *Taq* High Fidelity DNA Polymerase kit (Invitrogen, Carlsbad, CA, USA). The amplified fragment was TOPO-cloned into PCR8-GW-TOPO (Invitrogen) to produce an entry clone, PCR8-GW-PLK1, using the pCR8/GW/TOPO TA Cloning Kit (Invitrogen). Subsequently, the PLK1 coding sequence was transferred from the entry clone into the Gateway destination vector, pLenti6.3-TO-V5-DEST(Invitrogen), to generate the pLenti6.3-TO-PLK1-V5 construct, which encoded a tetracycline (Tet)-inducible PLK1, using an LR recombination reaction with LR Clonase™ II enzymes (Invitrogen).

The PCR8-GW-PLK1 construct was digested with *EcoR* I (New England Biolabs, Beverly, MA, USA) to remove the PLK1 fragment, and the cohesive end of the empty vector (EV) was self-ligated with T4 DNA ligase (New England Biolabs) to generate the PCR8-GW-TOPO-EV. The empty vector pLenti6.3-TO-V5-EV was then generated with the LR recombination reaction using PCR8-GW-TOPO-EV and pLenti6.3-TO-V5-DEST. The sequences of the chloramphenicol resistance gene and *ccd*B between *att*R1 and *att*R2 in pLenti6.3-TO-V5-DEST were replaced with the sequence between *att*L1 and *att*L2 in PCR8-GW-TOPO-EV during the LR recombination reaction. All constructs were confirmed by sequencing.

**Figure legend**

**Supplementary Figure 1­** Depletion of PLK1 expression induces cell cycle arrest and apoptosis in ESCC cells. KYSE150 and KYSE450 cells were transiently transfected with PLK1 siRNA or control non-silencing siRNA for 48 h. **(a)** The PLK1 expression level was assessed by Western blotting. β-actin was used as a loading control. (**b)** The cell cycle distribution were analyzed using ﬂow cytometry. Representative results are shown and cell cycle distribution was quantified. (**c)** Cell apoptosis was detected with an Annexin V-FITC double-staining assay and ﬂow cytometry. Representative results are shown and [percentage](javascript:void(0);) of apoptotic cells was plotted. **(b-c)** Data are mean ± SEM (n=3). *, *P* < 0.05; **, *P* < 0.01; ***, *P* < 0.001.
