# Supplementary figures and images for: Plumbagin inhibits the proliferation and survival of esophageal cancer cells by blocking STAT3-PLK1-AKT signaling
Source: Cell Death Dis. 2018 Jan 16;9(2):17. doi: 10.1038/s41419-017-0068-6 (PMC5833725; doi:10.1038/s41419-017-0068-6)

# Supplementary Figure 1

**a**

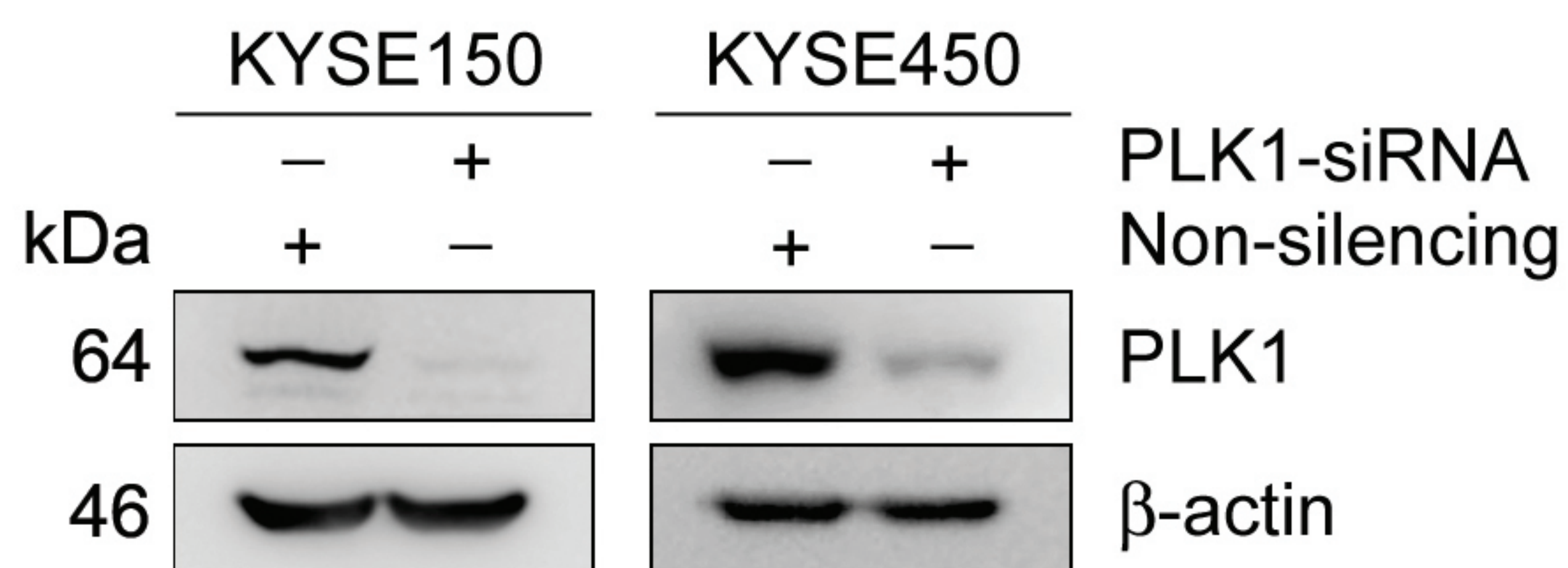

**b**

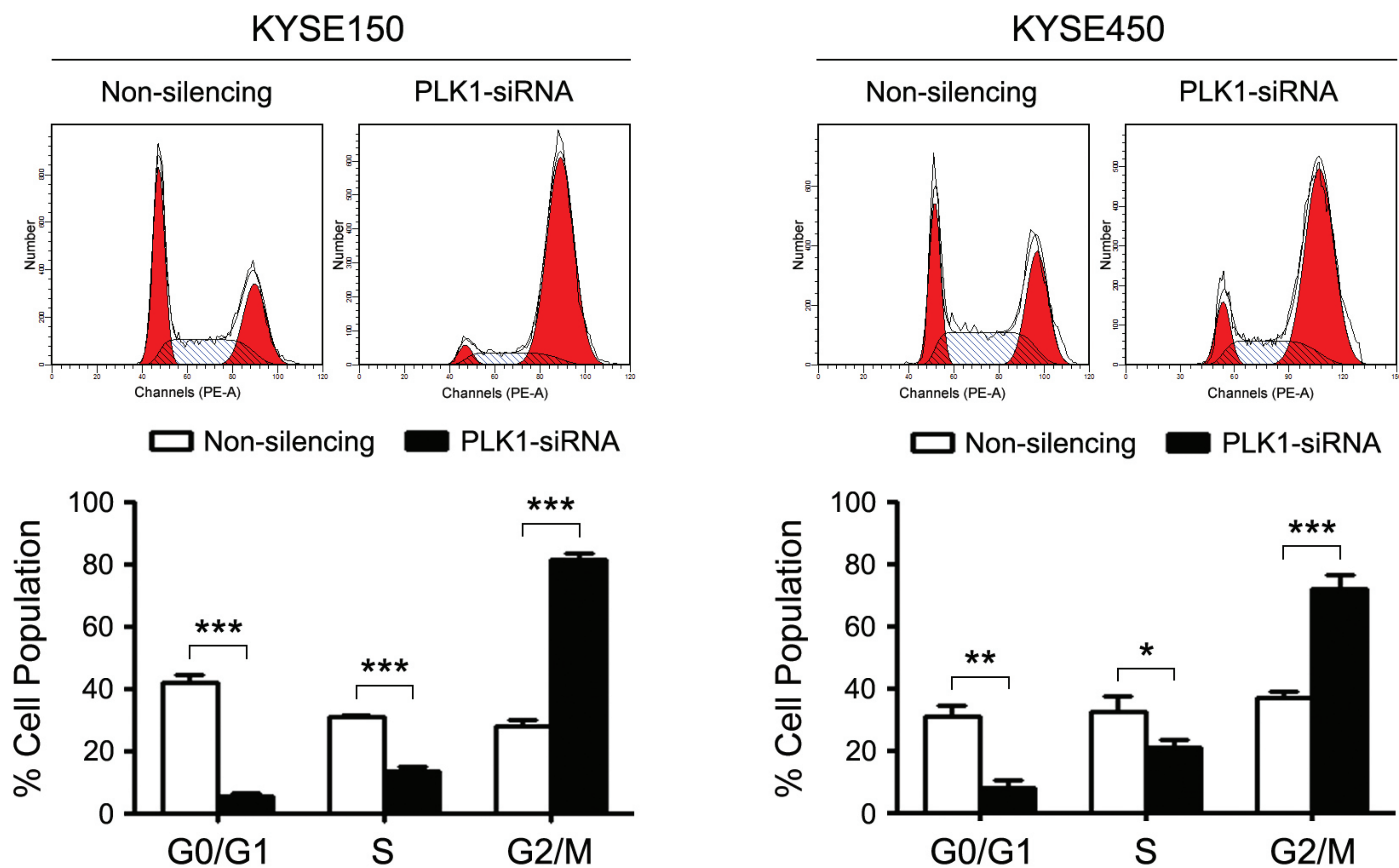

**c**

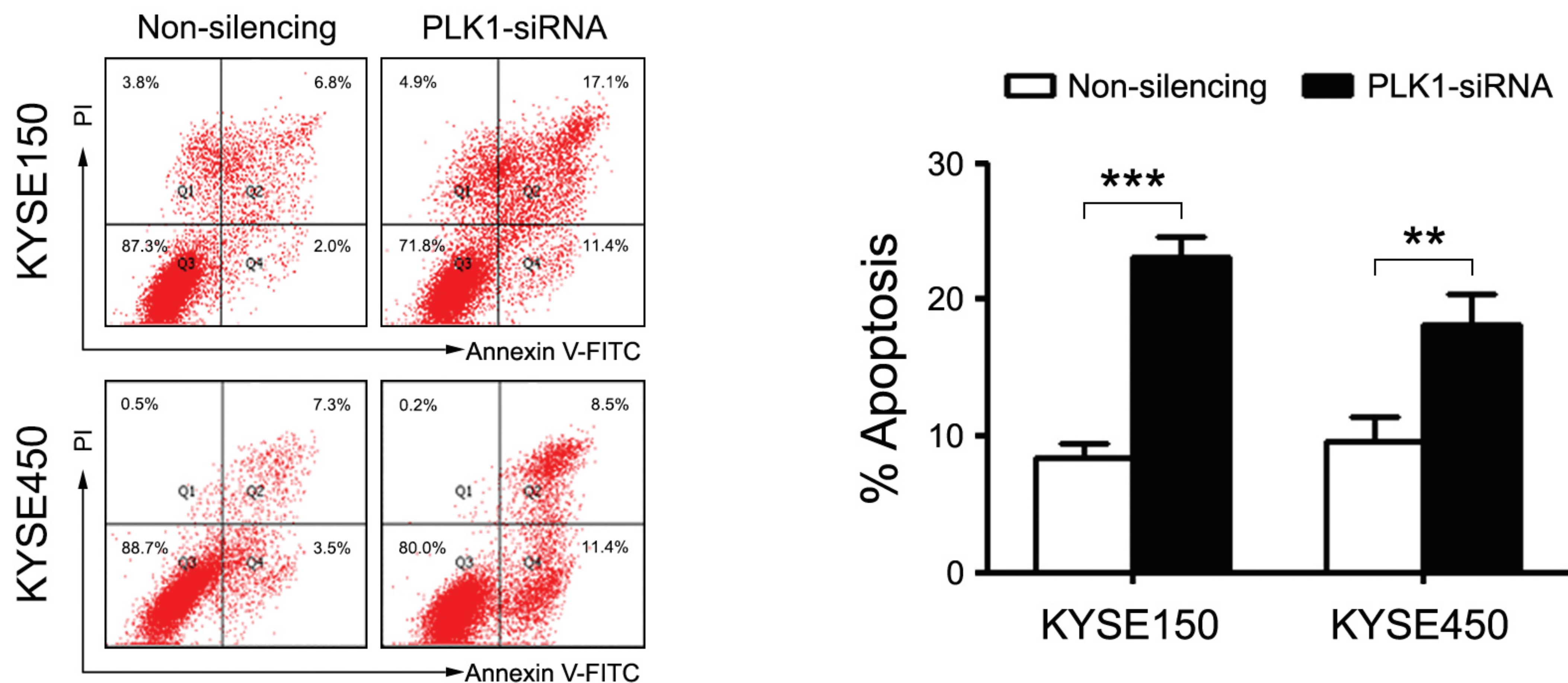

Supplement: Supplementary file 5 — Supplementary Figure 1 [file 41419_2017_68_MOESM5_ESM.pdf]
